# AUTOMINv1.0: an automation for minimization of Protein Data Bank files and its usage

**DOI:** 10.6026/97320630014525

**Published:** 2018-12-22

**Authors:** Rifat Nawaz Ul Islam, Debanjan Mitra, Parth Sarthi Sen Gupta, Sahini Banerjee, Buddhadev Mondal, Amal Kumar Bandyopadhyay

**Affiliations:** 1 Department of Zoology, The University of Burdwan, East Burdwan, West Bengal, India; 2 Department of Biotechnology, TheUniversity of Burdwan, East Burdwan, West Bengal, India; 3 Department of Chemistry, IISER Berhampur, Ganjam, Odisha, India; 4 Department of Biological Sciences, ISI, Kolkata, West Bengal, India; 5 Department of Zoology, Burdwan Raj College, East Burdwan,West Bengal, India

**Keywords:** Automation, minimization, shell-water, CHARMM, Salt-bridge, steric clash

## Abstract

Global minimal structure of protein/enzyme is energetically compromised that maintains an intricate balance between the rigidity and the flexibility. Such a state makes it interactive to its ligand molecules. Although protein data bank files (PDB) may have achieved the state, in many situations minimization has been crucial to overcome unwanted steric clashes, and other conformational strains. It is more so, when orthologous PDB structures that are intended in a given study, show variations in resolution, R-factor, shell-water contents, loop characteristics etc. Here, a fully automated procedure of minimization would be highly useful. AUTOMINv1.0 is such an automation of minimization that runs on any number of structure files with any number of chains in them along with the inclusion of selective/non-selective shell-waters interacting with polar and or non-polar atom-types of protein. Comparison of the mean binaryitems of salt-bridges of minimized and un-minimized structures (chains > 100) of nucleoside diphosphate kinase from mimi virus shows dramatic improvements in the earlier. Again, the mean steric clashes of 2AZ3.pdb are reduced upon minimization. Remarkably, the observed steric clashes between shell-waters and atom-types of protein are seen to be removed upon minimization. Taken together, AUTOMINv1.0 is an automation of minimization that finds applications in structural bioinformatics.

## Background

With the advent of protein's structure database, the concept of local-dataset-based proteomic scaled bioinformatics analyses is making rapid progress. Since candidate structures against a given UniProt ID and orthologous structures are many in the database, and since each of these structures has different state properties and experimental details, minimization in these cases seems to be crucial 1. Notably, in many cases, protein data bank (PDB) files are seen to have positive potential energy. Although on an individual basis such positive potential is considered relative, their inclusion in a local dataset (intended for bioinformatics studies) of PDBs with negative potential energy may produce confusing results. Here soft minimization essentially may bring these structures into an isoenergy state, largely by removing local steric-clashes and other conformational strains 2. Operationally, for a given chain, minimization is accomplished using a standard procedure 3. However, the procedure is far more complicated, when i] a PDB file possesses homomeric/heteromeric chains in them, ii] a family of PDB structures intended for a proteomicscaled- bioinformatics-studies and, iii] inclusion of crystallographic shell-waters are required. The fact that shellwaters are involved in many polar, non-polar interactions with protein's atom-groups, their inclusion in the process of minimization would improve the conformational state 4. Like earlier 5, 6, here we present an automation of minimization, AUTOMINv1.0, for all chains of a PDB and all PDBs present in a directory along with crystallographic shell-waters. The inclusion of selective shell-waters is made possible via mode dependent (mode: 1, 2 and 3) run-time parameters (sm, section-4). Taken together, AUTOMINv1.0 is a versatile automation of minimization for multi-meric PDB and PDBs in the presence of shell-waters, which seems to have potential applications in structural bioinformatics.

## Methodology

**Figure 1:**
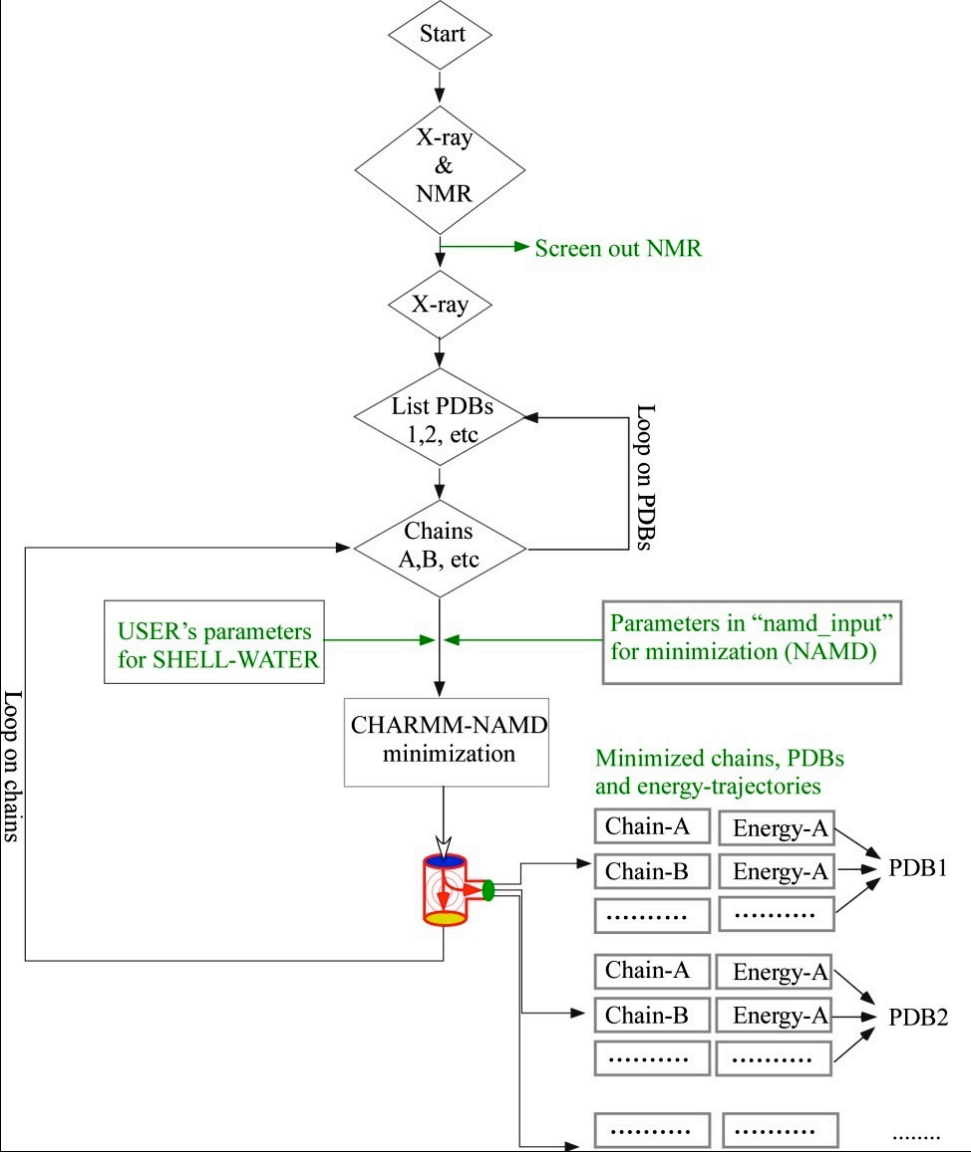
Flowchart for the functioning of AUTOMINv1.0. Upon start, the program lists only X-ray PDB files, even if the directory contains both X-ray and NMR files. Then, each PDB file is resolved into chains in that both protein and shell-waters are taken into account. Now parameters for shell-waters and NAMD configuration file (i.e. "namd_input") are to be set. AUTOMINv1.0 makes use of a CHARMM/VMD-NAMD minimization scheme. First, chain-specific minimization is performed. Once, all chains of a PDB file is completed, combined PDB file is written. Once a PDB file is completed, AUTOMINv1.0 does the same for next PDB and so on; until all PDBs are completed. Apart from minimized chain-specific and combined PDB files, the program also redirects their energy trajectories.

**AUTOMINv1.0** is automation for minimization of PDB file (X-ray format) with any number of chains in it and all PDBs present in a directory (sm, section-1). In doing so, it makes use of CHARMM topology, parameter files for solvation and minimization, which are accomplished using VMD and NAMD respectively 3. Although the latter two are not component of AUTOMINv1.0, these are needed to be installed for the functioning of the program. AUTOMINv1.0 facilitates automated minimization using i] distance dependent selective/all polar or ii] distance dependent selective/all polar and non-polar or iii] no crystallographic shell-waters (sm, section-4). The intended flexibility in setting parameters of NAMD configuration file (Figure 1) is done via "namd_input", which is kept under the disposal of users (Tables 1 and 2 - available with authors). Minimized chains, chains-combined-PDB and energy trajectories of minimization are redirected as outputs (Figure 1).

## Program input:

**AUTOMINv1.0** needs three inputs. First, it requires PDB files in the directory. Second, "namd_input" contains 28 parameters of which first 5 are set by AUTOMINv1.0. Rest (6-28 parameters) can be flexibly set by users. The input file is a typical NAMD configuration file, which the program uses for automated minimization (Tables 1 and 2 - available with authors). Third, type-specific (polar/non-polar) and distance-specific (selective/all/none) crystallographic shell-waters can be included in the minimization by AUTOMINv1.0 (sm, section-3 and 4).

## Program output:

**Figure 2:**
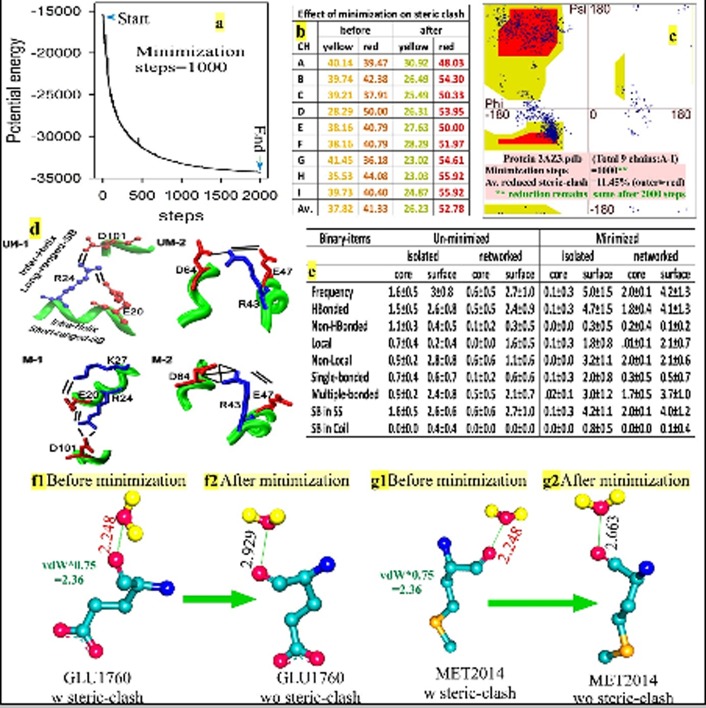
Automated minimization of chains of protein (a) using AUTOMINv1.0. Minimization eliminates average steric clashes (b and c), improves binary items of salt-bridges and its energetics (d and e). The steric-clashes in shell-water and protein interactions (f1 and g1) are also eliminated on minimization (f2 and g2).

For a given PDB file, AUTOMINv1.0 redirects three types of outputs. Chain-specific and all-chains combined minimized PDB files along with their minimization energy profiles are obtained as outputs (Figure 2). While minimized PDBs are suitable for any type of post-run analyses, energy profiles allow the user to check the level of minimization and quality of run (Figure 2). Orthologous structure files with a number of chains in each file may be required to be studies using the mean observations. Although for a given structure with many chains, the details on expression system, crystallization conditions, R-factors, and ground-state potential energy are identical, candidate PDBs of a given family have variations in these details, which could largely be improved upon minimization. Such mass-scaled minimization could suitably be achieved by the use of a fully automated procedure. AUTOMINv1.0 is such a procedure that automates minimization of all chains in a PDB and all PDBs in a directory in the presence of shell-water. Few of the analysis are presented in Figure 2. 2AZ3.pdb is a salt adapted nucleotide diphosphate kinase (NDK) from Halobacterium salinarum with its saltdependent properties as halophilic ferredoxin 7, with pI 4.3 and GRAVY -0.6 [6]. The distribution of phi and psi of its 9 chains for the red-region of the Ramachandran plot (free from steric-clash) are shown in Figure 2 before and after minimization 8. It is seen that chain specific and average steric-clashes are remarkably reduced in the latter case. Further, the mean observations on binary-items of salt-bridges of 26 structures with a total of 102 chains of NDK from mimivirus 9-12 show an improvement upon minimization (Figure 2). Remarkably, networked salt bridges that reduced isolated charges and desolvation cost and thereby promote stability 11,13 show their increased level in the core-region, upon minimization (sm, section-2). Minimization also promotes extended networked saltbridges (Figure 2, M1) in comparison to un-minimized one (Figure 2, UM1). It also improves the geometry of salt bridges (Figure 2, M2) than the un-minimized form (Figure 2d, UM2). Further, the steric clash between shell-water and protein is disappeared in a minimized state (Figure 2, f1 & f2 and g1 & g2).

## Caveats and future development:

**AUTOMINv1.0** is interpreted by AWK/GAWK programming language, which can preferably run in CYGWIN 32bit OS in a Windows environment. It is compiled using AWKA (URL: http://awka.sourceforge.net/index.html) Presently we are actively engaged in developing a web interface to integrate AUTOMINv1.0 and other structure related programs of our laboratory such as ADSBET 14 and COSURIM 15 such that their availability could reach to academic users within a unique web service.

## Conclusion

**AUTOMINv1.0,** which is compiled using AWKA (URL: http://awka.sourceforge.net/index.html), is interpreted by AWK/GAWK programming language in CYGWIN 32bit OS in a Windows environment. Protein structures of i] homologous families and ii] identical UniProt IDs can suitably be optimized using the program prior to some comparative bioinformatics studies.

## References

[1] Xu D, Zhang Y (2011). Biophys. J..

[2] Ramachandran S (2011). Proteins Struct. Funct. Bioinf..

[3] Pedretti A (2004). J. Comput.-Aided Mol. Des..

[4] Teeter MM (1991). Annu. Rev. Biophys. Chem..

[5] Banerjee S (2015). Bioinformation.

[6] Gupta PS (2014). Bioinformation.

[7] Bandyopadhyay AK, Sonawat HM (2000). Biophys. J..

[8] Gupta PS (2013). American Journal of Bioinformatics Research.

[9] Gupta PS (2014). Bioinformation..

[10] Gupta PS (2015). Bioinformation..

[11] Nayek A (2014). Plos one..

[12] Kumar S, Nussinov R (1999). J. Mol. Biol..

[13] Nayek A (2015). Bioinformation.

[14] Nayek A (2015). International Journal of Institutional Pharmacy and Life Sciences.

[15] Gupta PS (2017). International Journal of Engineering Science and Technology.

